# Reovirus-induced cell-mediated immunity for the treatment of multiple myeloma within the resistant bone marrow niche

**DOI:** 10.1136/jitc-2020-001803

**Published:** 2021-03-19

**Authors:** Louise M E Müller, Gemma Migneco, Gina B Scott, Jenny Down, Sancha King, Basem Askar, Victoria Jennings, Babatunde Oyajobi, Karen Scott, Emma West, Christy Ralph, Adel Samson, Elizabeth J Ilett, Munitta Muthana, Matt Coffey, Alan Melcher, Christopher Parrish, Gordon Cook, Michelle Lawson, Fiona Errington-Mais

**Affiliations:** 1 Division of Haematology and Immunology, Leeds Institute of Medical Research, University of Leeds, Leeds, UK; 2 Sheffield Myeloma Research Team, Department of Oncology and Metabolism, University of Sheffield, Sheffield, UK; 3 Division of Radiotherapy and Imaging, Institute of Cancer Research, London, UK; 4 Cancer Therapy and Research Center, The University of Texas Health Science Center at San Antonio, San Antonio, Texas, USA; 5 Oncolytics Biotech Inc, Calgary, Alberta, Canada; 6 Department of Haematology, St James's University Hospital, Leeds, UK; 7 Leeds Institute of Clinical Trials Research, University of Leeds, Leeds, UK

**Keywords:** oncolytic viruses, adaptive immunity, immunity, innate immunity, immunotherapy

## Abstract

**Background:**

Multiple myeloma (MM) remains an incurable disease and oncolytic viruses offer a well-tolerated addition to the therapeutic arsenal. Oncolytic reovirus has progressed to phase I clinical trials and its direct lytic potential has been extensively studied. However, to date, the role for reovirus-induced immunotherapy against MM, and the impact of the bone marrow (BM) niche, have not been reported.

**Methods:**

This study used human peripheral blood mononuclear cells from healthy donors and in vitro co-culture of MM cells and BM stromal cells to recapitulate the resistant BM niche. Additionally, the 5TGM1-Kalw/RijHSD immunocompetent in vivo model was used to examine reovirus efficacy and characterize reovirus-induced immune responses in the BM and spleen following intravenous administration. Collectively, these in vitro and in vivo models were used to characterize the development of innate and adaptive antimyeloma immunity following reovirus treatment.

**Results:**

Using the 5TGM1-Kalw/RijHSD immunocompetent in vivo model we have demonstrated that reovirus reduces both MM tumor burden and myeloma-induced bone disease. Furthermore, detailed immune characterization revealed that reovirus: (i) increased natural killer (NK) cell and CD8^+^ T cell numbers; (ii) activated NK cells and CD8^+^ T cells and (iii) upregulated effector-memory CD8^+^ T cells. Moreover, increased effector-memory CD8^+^ T cells correlated with decreased tumor burden. Next, we explored the potential for reovirus-induced immunotherapy using human co-culture models to mimic the myeloma-supportive BM niche. MM cells co-cultured with BM stromal cells displayed resistance to reovirus-induced oncolysis and bystander cytokine-killing but remained susceptible to killing by reovirus-activated NK cells and MM-specific cytotoxic T lymphocytes.

**Conclusion:**

These data highlight the importance of reovirus-induced immunotherapy for targeting MM cells within the BM niche and suggest that combination with agents which boost antitumor immune responses should be a priority.

## Background

Multiple myeloma (MM) is a tumor of terminally differentiated plasma cells which expand in the bone marrow (BM). MM results in the onset of myeloma-induced bone disease (MBD) due to increased bone resorption by osteoclasts and loss of osteoblast function. MBD is one of the many debilitating features of MM which results in bone pain and frequent fractures.^1^ The worldwide incidence of MM is ~114 000 cases per year and numbers are expected to increase with an aging population.^2^ Currently, MM remains an incurable disease in need of novel treatment strategies that are safe and well-tolerated.^2^

The BM microenvironment has a diverse cellular composition, including haematopoietic stem cells, mesenchymal stem cells, BM stromal cells (BMSCs; fibroblasts and epithelial cells) and immune cells. Moreover, the BM niche provides a range of cytokines, chemokines and growth factors which all contribute to MM progression and therapy resistance.^3 4^ Oncolytic viruses (OVs) preferentially infect and kill malignant cells, and use multiple mechanisms to eradicate tumor cells, including engagement of both innate and adaptive antitumor immune responses.^5 6^ Oncolytic virotherapy has gained increasing attention over recent years following the Food and Drug Administration approval of Talimogene laherparepvec (T-Vec), a modified herpes simplex virus-1 (HSV-1), which encodes granulocyte-macrophage colony-stimulating factor (GM-CSF), for the treatment of advanced melanoma. Unfortunately, in comparison to solid malignancies, OVs are relatively under investigated in the context of hematological malignancies (HM) and, as such, their progression to clinical trials has been limited; to date, phase I clinical trials in MM have been reported for reovirus, measles virus (MV) and vesicular stomatitis virus.^7^

Reovirus is a double-stranded RNA virus that uses junctional adhesion molecule A (JAM-A) for viral entry,^8^ kills neoplastic cells through both apoptotic and non-apoptotic mechanisms^9 10^ and can use activated RAS signaling for replication and oncolysis^9^; significantly, JAM-A is overexpressed in MM^11^ and mutated *Ras* is associated with MM progression.^12^ In accordance with this, the direct lytic potential of reovirus against MM has been reported^13^ and early phase clinical trials have been carried out using reovirus type 3 *Dearing* strain (T3D; pelareorep).^14^ Pelareorep was well-tolerated in patients with MM, and only low-grade adverse effects were reported.^14 15^

Innate and adaptive immunity are important for reovirus efficacy and the secretion of type I interferon (IFN) is a key component of the innate immune response.^16^ IFN-α is important for natural killer (NK) cell activation in response to reovirus,^16^ although a range of other pro-inflammatory cytokines mediate the recruitment of NK cells and dendritic cells (DC) to the tumor.^17^ Reovirus also enhances the ability of DC to present tumor-associated antigens (TAA) for priming of tumor-specific cytotoxic T lymphocytes (CTLs).^18^ Harnessing reovirus-induced antitumor immunity has the potential to induce immunological memory and long-term cancer remission in patients with MM.

Unfortunately, despite promising preclinical data, single-agent reovirus treatment was ineffective at treating relapsed/refractory MM. This dichotomy highlights the need for us to better understand reovirus–host interactions, in particular, which reovirus effector mechanisms are induced within the BM niche and which have the capacity to eradicate MM cells that are supported by the tumor microenvironment. Once identified, key effector mechanisms can be prioritized for the development of effective combination strategies designed to boost reovirus efficacy in clinical trials. Therefore, this study aimed to characterize the immune response to reovirus in a BM niche and identify key effector mechanisms underpinning reovirus efficacy.

## Methods

### 5TGM1 in vivo model

C57BL/KaLw/RijHSD mice were purchased at age 5–10 weeks from the St James’s Biomedical Services, University of Leeds. Mice were housed in individually ventilated, positive pressure ISOcages (five mice per cage). All mice were subjected to a regulated daylight cycle, had access to water, standard mouse feed and nesting material and were pathogen free. All animals were monitored daily and any mice exhibiting hind limb paralysis (HLP), or other distress, were euthanized by cervical dislocation.

#### Reovirus therapy experiment

Both female and male mice aged 5–8 weeks were used and all groups were randomized based on age and sex. Mouse group numbers were ascertained using the power calculation formula (2(SD)^2^×f(α,β)/Δ^2^). The α (significance level) was 0.05, β (power level) was 90% and both Δ (least practicable difference between groups) and SD were taken from a pilot study to determine reovirus efficacy. This gave rise to the following power calculation (2(8.33)^2^×10.5/15^2^=6.47) and seven mice/group were used for all subsequent in vivo experiments.

On day 0, mice were injected with 2×10^6^ bone-homing 5TGM1 cells (a kind gift from Prof Oyajobi)^19^ in 100 µL phosphate-buffered saline (PBS) via the lateral tail vein. On day 7–9, reovirus or control (PBS) treatment was initiated with 3 weekly injections (Monday/Wednesday/Friday) of 2×10^7^ plaque-forming units (PFU)/mL reovirus in 100 µL PBS or 100 µL PBS alone, respectively. Treatment continued until the development of HLP in PBS-treated mice (20–27 days) when all mice were sacrificed and tissues were harvested for assessment of tumor burden, bone analysis and immunophenotyping (see online supplemental tables S1 and S2 for details of the flow cytometry antibodies used).

10.1136/jitc-2020-001803.supp1 Supplementary data

#### Assessment of MBD by micro-CT

To assess bone disease, tibiae were analyzed by micro-CT (μCT) using a SkyScan 1272 ex vivo μCT scanner at 50 kV and 200 µA, using an aluminum filter of 0.5 mm and pixel size of 4.3 µm^2^, as described previously.^20^ Bone volume (BV)/total volume, trabecular number, trabecular thickness, cortical thickness, lesion area and lesion number parameters were then assessed as described previously^20^ and according to standard guidelines.^21^

#### SYBR Green reovirus RT-qPCR

One-step quantitative PCR (qPCR) reactions were prepared in triplicate in 96-well MicroAmp Optical Reaction plates (Applied Biosystems) using the *Power* SYBR Green RNA-to-C_T_ *1-Step* Kit (ThermoFisher Scientific); 20 µL reaction mixes included: 0.3 μg RNA, 1× *Power* SYBR Green RT-PCR Mix, 1× RT Enzyme Mix, 0.5 μM of σ3 forward and reverse primers and RNase-free water. A 10fold serial dilution of reovirus RNA, isolated from stock reovirus, was included for quantification of reovirus RNA. Thermal cycling was performed on the QuantStudio 5 Real-Time PCR System (Applied Biosystems).

### Cell culture

NCI-H929, U266B, JIM3 and OPM2 human MM cell lines, KG-1 (an acute myeloid leukemia cell line) and HS-5 (fibroblast-like) and HS-27 (epithelial-like) BMSCs were obtained from the American Type Culture Collection and cultured in RPMI-1640 (Sigma-Aldrich) supplemented with 10% fetal calf serum (FCS; Gibco). All cells were routinely tested for mycoplasma and were free from infection. *Isolation of peripheral blood mononuclear cells (PBMC*): healthy donor blood was obtained from leukocyte apheresis cones supplied by the National Health Service Blood and Transplant unit. PBMC were isolated using Lymphoprep (Fresenius-Kabi) and seeded at 2×10^6^ cells/mL in RPMI-1640 supplemented with 10% FCS.

#### CellTracker staining

A 5 mM stock solution of Cell Tracker Green (CTG) CMFDA fluorescent dye (Invitrogen) was prepared in dimethyl sulfoxide and a 2.5 μM working dilution was prepared in prewarmed serum-free RPMI-1640. Cells were stained at 10^6^/mL for 30 min at 37°C and washed three times in complete RPMI-1640 before use.

#### MM and BMSC co-cultures

HS-5 and HS-27 BMSCs cells were allowed to adhere overnight and human MM cell lines (NCI-H929, U266B and JIM3) were stained with CTG and added to adherent BMSCs at a 1:1 ratio in an equal volume of fresh medium. Cells were co-cultured for 24 or 48 hours before use (online supplemental figure 1).

10.1136/jitc-2020-001803.supp2 Supplementary data

#### PBMC-conditioned medium

PBMC were seeded at 2×10^6^ cells/mL and treated with 0.1 or 1 PFU/cell reovirus, or mock treated with PBS. After 48 hours incubation, cells were removed by centrifugation (400× *g* for 5 min) and the supernatant was sterile filtered using a 0.2 μm syringe filter (Millex, Merck Millipore) and stored at −20°C. To inactivate reovirus, conditioned medium (CM) was UV-irradiated using a C-1000 UV Crosslinker for 2 min (1200 μJ/cm^2^) in 2 mL aliquots in an open 6-well plate (online supplemental figure S2). To remove extracellular vesicles, PBMC-CM was filtered using Amicon Ultra-15 centrifugal filters. Reovirus T3D strain was provided by Oncolytics Biotech Inc.

### MTS assay

A total of 5×10^4^ MM cells were seeded in 50 μL/well and 50 μL CM was added in triplicate (1:1 dilution in fresh medium). Plates were incubated for 96 hours before MTS reagent (tetrazolium dye, Abcam) was added. The optical density was measured at 450 nm using a Multiscan EX microplate reader (ThermoFisher Scientific).

### Priming of tumor-specific CTLs

#### Generation of human myeloid-derived DC

CD14^+^ monocytes were isolated from PBMC using magnetic cell sorting (Miltenyi Biotec). CD14^+^ cells were cultured at 8×10^5^/mL in RPMI-1640, 10% FCS, 800 U/mL GM-CSF (MBL International) and 500 U/mL interleukin-4 (BioLegend) for 5 days to obtain immature DC (iDC).

#### Generation of tumor-specific CTLs

Tumor cells were either left untreated, or treated with 1 PFU/cell reovirus for 24 hours. Immature DC were loaded with tumor cells (±reovirus treatment) at a 3:1 tumor cell:DC ratio for 48 hours and tumor-loaded DC were cultured with autologous PBMC at a 1:20-1:30 DC:PBMC ratio in CTL medium.^6^ Cells were incubated for 7 days at 37 ºC and re-stimulated with tumor-loaded iDC (±reovirus treatment) for a further 6 days before use.

#### Peptide pool stimulation of primed CTLs

PepTivator Peptide Pools (Miltenyi Biotec) consisting of 15-mer sequences of amino acids with 11 amino acids overlap covering the PRAME, Mucin-1 and MAGE-A1 proteins were used; PepTivator Peptide Pools were stored at 30 nmol/mL at −80°C. For TAA stimulation, 2×10^6^ autologous CD14^+^ were incubated with appropriate peptide pools for 60 min at 37°C at a final concentration of 6 nmol/mL. CD14^+^ cells (±peptide) were then co-cultured with autologous CTLs at a 2:1 ratio and examined for intracellular IFN-γ by flow cytometry.

### Flow cytometry

All flow cytometry was performed and analyzed using a 2-laser Attune Acoustic Focusing Cytometer (Applied Biosystems) or a 4-laser CytoFLEX S (Beckman Coulter) and analyzed using appropriate software. Antibodies details are provided in online supplemental tables S1 and S2.

#### LIVE/DEAD assay

Cells were harvested, washed in 1 mL PBS and stained with LIVE/DEAD Fixable Yellow Dead Cell Stain (Invitrogen; 500 μL diluted 1:1000 in PBS) for 30 min in the dark. Samples were washed with PBS and then fixed with 300 μL 1% paraformaldehyde (PFA) in PBS.

#### NK cell and CTL CD107 degranulation assays

Effector lymphocytes were incubated alone or with target tumor cells at a 2:1 ratio. After 1 hour at 37 ºC, anti-CD107a, anti-CD107b and either anti-CD3/CD8 (CTL population) or anti-CD3/CD56 (NK cells) were added, along with 10 µg/mL brefeldin A (BioLegend). Samples were then incubated for a further 4 hours at 37 ºC, washed with fluorescence-activated cell sorting (FACS) buffer (PBS, 1% FCS and 0.1% sodium azide) and fixed in 1% PFA until acquisition.

#### Intracellular IFN-γ staining

Primed PBMC were co-cultured with tumor targets or peptide-loaded CD14^+^ cells and treated as described for the CD107 degranulation assay (above), without the addition of CD107 antibodies. Following fixation in 1% PFA overnight, cells were washed in FACS buffer, permeabilized in 0.3% saponin (Sigma-Aldrich) in FACS buffer for 15 min (room temperature) and then stained with a fluorescein isothiocyanate-conjugated IFN-γ antibody prior to acquisition.

#### Flow cytometry-based killing assay

Target cells were either stained with CTG immediately prior to the assay, or for target cells co-cultured on BMSCs for 48 hours CTG staining occurred prior to co-culture set up. Target MM cells were harvested by washing with PBS+2.5 mM EDTA and co-cultured with effector cells (CTLs and NK cells) at a 25:1 effector:target ratio for 5 hours at 37°C. Cells were subsequently stained with the yellow LIVE/DEAD and fixed in 1% PFA.

### ^51^Chromium release assay

Target cells were labeled with 100 μCi ^51^Cr (PerkinElmer) per 10^6^ cells for 1 hour at 37°C. Effector cells were harvested and co-cultured with ^51^Cr-labeled target cells at different effector:target ratios. Spontaneous release was established using target cells in medium and maximum release was obtained using 1% Triton-X (Sigma-Aldrich). For CTL assays, unlabeled K562 and Daudi target cells were included to mitigate NK cell and lymphokine-activated killer cell activity. Following the 4 hour incubation, 50 μL of cell-free supernatant was transferred to a LumaPlate (PerkinElmer) and the level of ^51^Cr was then measured using a Microbeta^2^ scintillation counter (PerkinElmer). The per cent cell lysis was calculated as below (cpm: counts per minute):

$$\%\;lysis=100\;\times \frac{sample\;cpm-spontaneous\;cpm}{maximum\;cpm-spontaneous\;cpm}$$

### Statistical analysis

Statistical analysis was performed using Graphpad Prism V.7.0 (GraphPad Software, La Jolla, California, USA). P values were calculated using either Student’s t-test with two-tailed distribution or one-way or two-way analysis of variance. Results were considered significant if p value was <0.05. Pearson’s r was calculated to evaluate correlation.

## Results

### In vivo efficacy of reovirus

**Figure 1:**
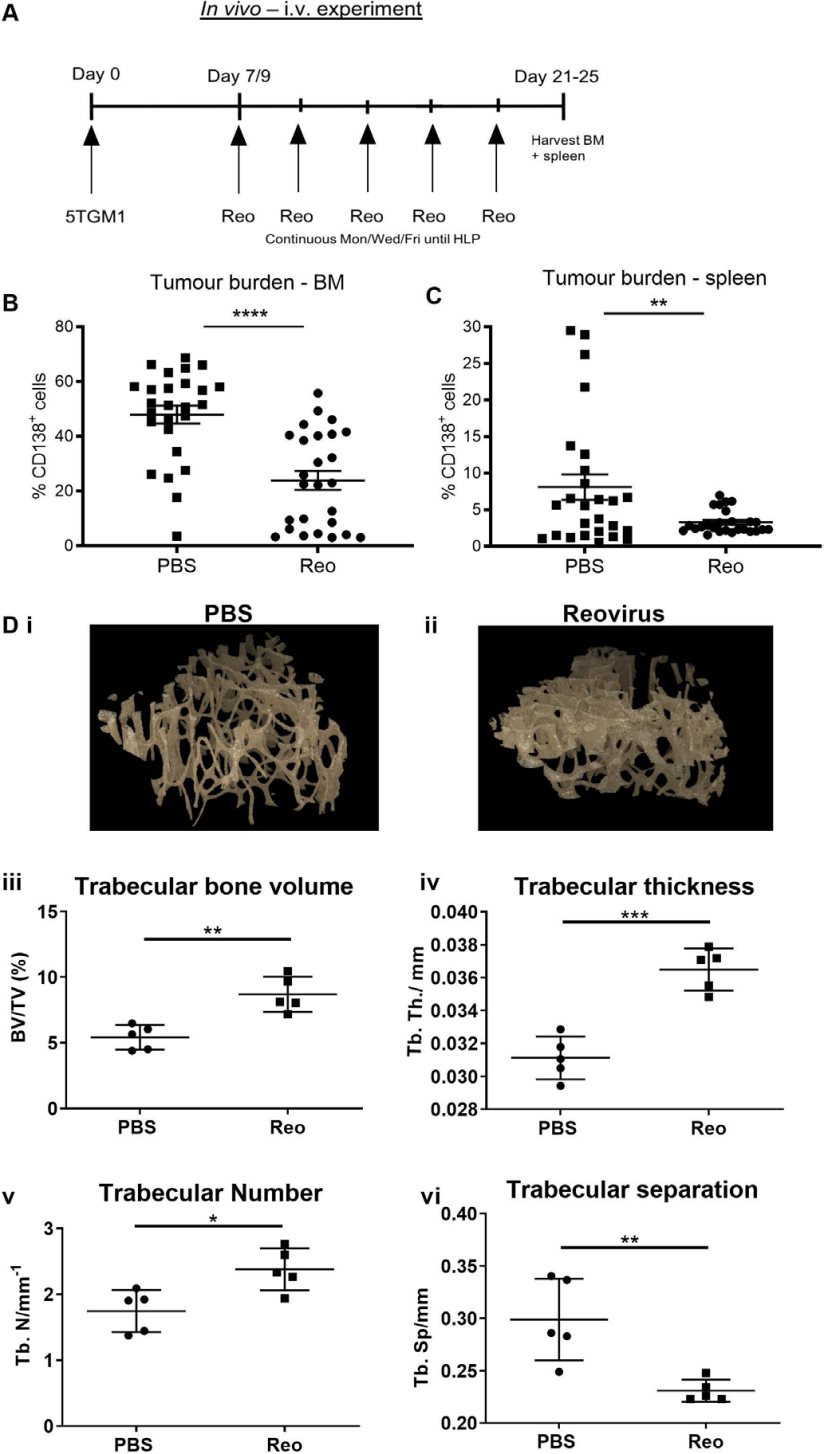
Reduction in tumor burden and prevention of myeloma bone disease following intravenous reovirus treatment. (A) Schematic in vivo treatment schedule. Mice were injected with 2×10^6^ 5TGM1 cells intravenously on day 0. Reovirus (Reo) therapy (2×10^7^ plaque-forming units) or phosphate-buffered saline (PBS) was started on day 7–9 and continued three times weekly until development of hind limb paralysis in PBS mice (day 21–25). Following sacrifice, the bone marrow (BM) and spleen were harvested. Tumor burden in the BM (B) and spleen (C) after PBS or reovirus treatment was determined as the percentage of CD138^+^ cells (n=26; amalgamated results from repeat experiments). (D) Μicro-CT images after treatment with PBS (i) or reovirus (ii). Analyses of trabecular bone volume (iii), thickness (iv), number (v) and separation in tibias (vi) of 5TGM1-bearing mice (3 weeks post-tumor cell injection) treated with PBS or reovirus (Reo). Asterisk (*) denotes statistical significance and error bars indicate SEM. *P<0.05, **p<0.01, ***p<0.001, ****p<0.0001.

In vivo efficacy of reovirus following intravenous administration: the stroma-independent 5TGM1 MM subclone, established in immunocompetent C57Bl/KaLw/RijHSD mice mimics human MM by secreting paraprotein, causing hypercalcaemia, and inducing MBD.^22^ After establishment of 5TGM1 tumors (~1 week postinjection), mice received reovirus treatment or PBS (intravenous) until the development of HLP in PBS control groups (figure 1A). Following reovirus treatment, a significant reduction in tumor burden in the BM and spleen was observed, compared with the PBS control (figure 1B, C). Significantly, decreased tumor burden was also associated with reduced MBD as demonstrated by the prevention of trabecular bone loss, where higher levels of trabecular BV, trabecular thickness and trabecular number were observed, in accordance with reduced trabecular separation (figure 1D).

#### Characterization of immune effector cells following reovirus treatment

**Figure 2:**
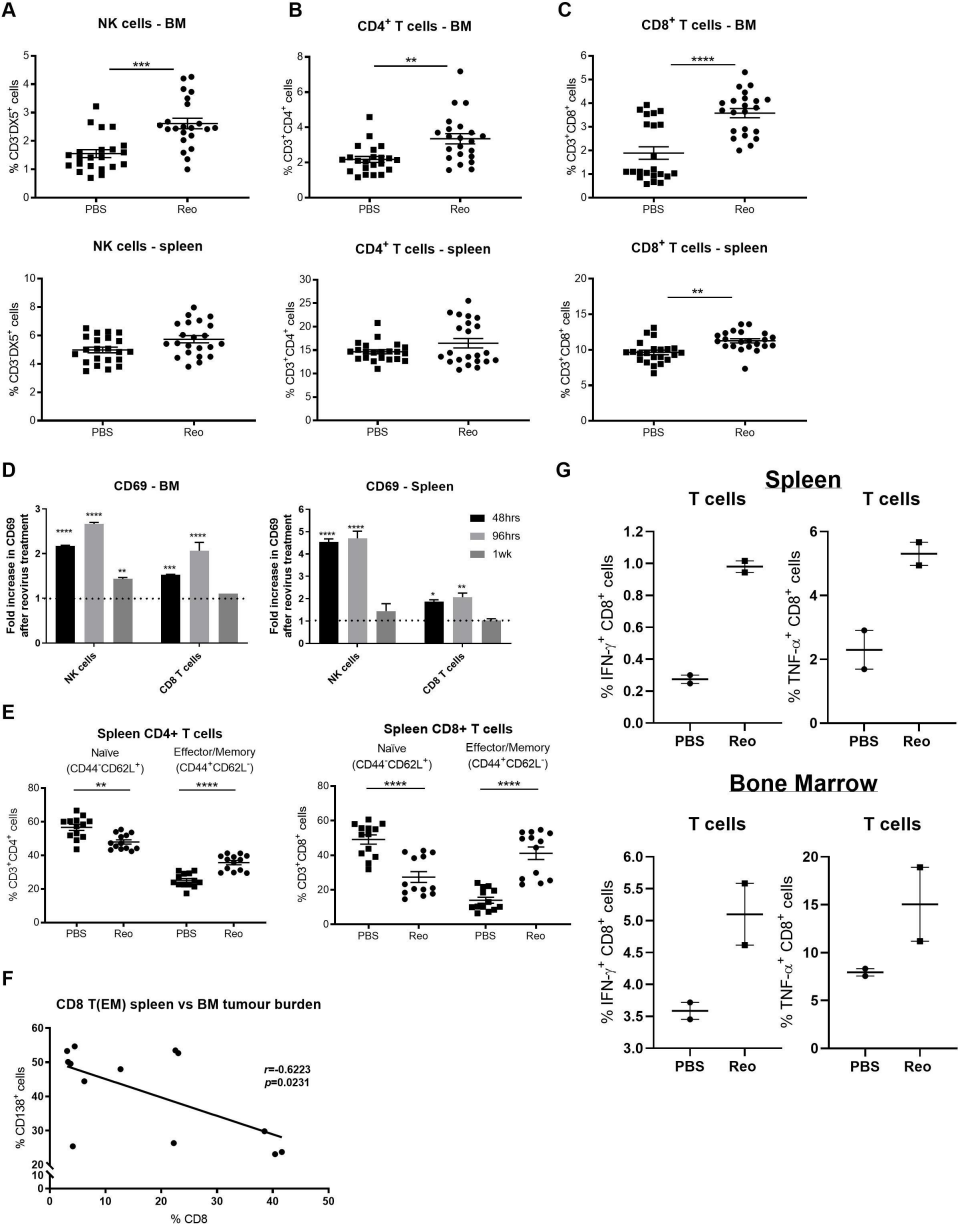
In vivo characterization of natural killer (NK) cells and T cells following intravenous reovirus treatment. The percentage of CD3^−^DX5^+^ NK cells (A), the percentage of CD3^+^CD4^+^ T cells (B) and the percentage of CD3^+^CD8^+^ T cells (C) in the bone marrow (BM) and spleen (n=22 per group) at the time of sacrifice. (D) Fold increase in CD69 expression on CD3^-^DX5^+^ NK cells and CD3^+^CD8^+^ T cells compared with phosphate-buffered saline (PBS)-treated mice. Data were collected 48 hours, 96 hours and 1 week after the first reovirus injection (n=3 mice/time point). (E) At sacrifice, the percentage of CD4^+^-naïve and CD8^+^-naïve T cells (CD44^−^CD62L^+^) and effector/memory T cells (T_EM_, CD44^+^CD62L^−^) were quantified in the BM and spleen (n=13). Error bars indicate SEM and asterisk (*) denotes statistical significance. (F) Correlation of the percentage of CD138^+^ tumors cells within the bone marrow with the percentage of CD8^+^ T_EM_ in the spleen. Pearson’s r value is shown. (G) Ninety-six hours post-treatment, splenocytes (top) and BM cell (bottom) were isolated and co-cultured with 5TGM1 cells ex vivo for 4 hours in the presence of brefeldin A. The percentage of IFN-γ^+^CD8^+^ and TNF-α^+^CD8^+^ T cells were quantified by flow cytometry. *P<0.05, **p<0.01, ***p<0.001, ****p<0.0001.

We established that 5TGM1 cells were susceptible to reovirus-direct oncolysis in vitro (online supplemental figure S3A). However, the fact that we were unable to detect reovirus in the BM of reovirus-treated animals by RT-qPCR on termination of the experiment despite repeated reovirus administration (online supplemental figure S3B), suggested that reovirus may exert its effects through immune-mediated mechanisms. To explore this, we examined the proportion and activation status of NK cells, CD4^+^ T cells and CD8^+^ T cells in the BM and spleen following systemic reovirus administration. Following reovirus treatment, NK cells and CD4^+^ T cells were more prevalent in the BM (figure 2A, B, respectively) and the proportion of CD8^+^ T cells was increased in both the BM and spleen (figure 2C). On assessment of CD69 expression (an early marker of lymphocyte activation) on NK cells and CD8^+^ T cells, increased expression was observed in both the BM and spleen 48 and 96 hours post-treatment; CD69 expression was reduced to baseline 1 week postreovirus treatment, with the exception of NK cells within the BM (figure 2D).

10.1136/jitc-2020-001803.supp4 Supplementary data

To provide further characterization, T cells were phenotyped to: (i) examine changes in programmed cell death protein 1 (PD-1) expression as a further indicator of immune activation/control and (ii) quantify the proportion of naïve T cells (CD44^−^CD62L^+^; indicating reconstitution of the normal BM) and effector T cells (T_EM_, CD44^+^CD62L^−^; indicating an antitumor and/or antiviral T cell response).^23^ A significant increase in PD-1 expression on both CD4^+^ and CD8^+^ T cells was observed in the spleen following reovirus treatment (online supplemental figure S3C); enhanced numbers of CD4^+^ and CD8^+^ T_EM_ cells were also observed, concordant with a decrease in naïve T cells (figure 2E). Moreover, in reovirus-treated mice there was a significant negative correlation between the percentage of CD8^+^ T_EM_ cells in the spleen and the percentage of CD138^+^ tumor cells in the BM (figure 2F; p=0.023, Pearson’s r=−0.62), suggesting a potential role for adaptive T cell antitumor immunity for the eradication of MM cells. In support of this, preliminary data demonstrated an enhanced proportion of IFN-γ-producing and TNF-α-producing CD8^+^ T cells following in vitro stimulation with 5TGM1 cells (figure 2G), suggesting that reovirus-treated animals had a greater percentage of 5TGM1-specific CD8^+^ T cells in the BM and spleen (96 hours post-treatment initiation).

Overall, these data demonstrate the in vivo efficacy of reovirus against MM and associated MBD, and suggest a role for reovirus-induced innate and adaptive antitumor immunity for its therapeutic efficacy. However, establishing the importance of each of these mechanisms in the context of human disease is essential to inform the future development of reovirus therapy.

### Modeling the human BM niche in vitro

**Figure 3:**
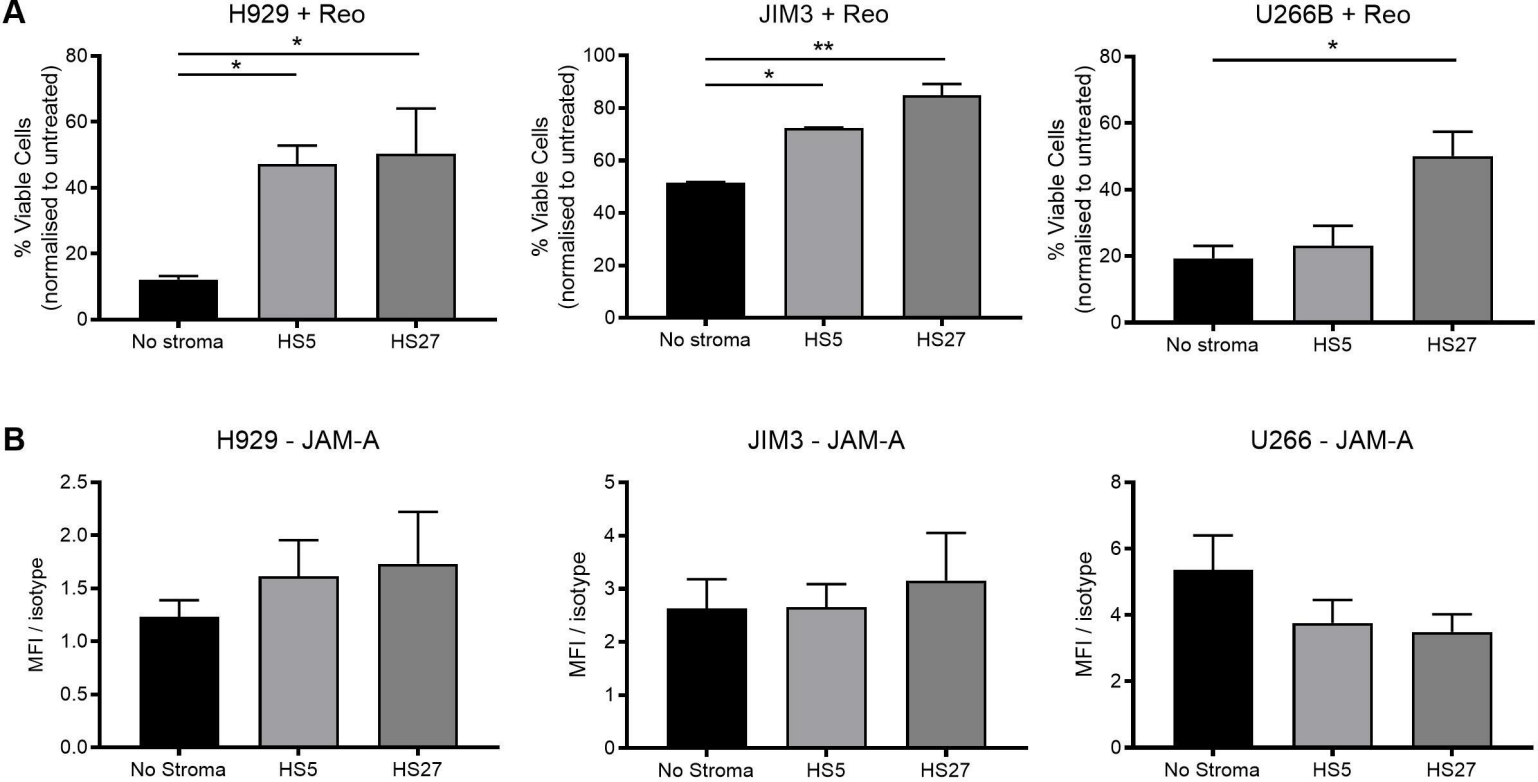
Co-culture of multiple myeloma (MM) cells with bone marrow stromal cells (BMSCs) inhibits reovirus-direct oncolysis. H929, JIM3 and U266B cells (labeled with Cell Tracker Green (CTG)) were either treated alone, or co-cultured with HS-5 or HS-27 BMSCs at a 1:1 ratio for 24 hours. (A) Cells were treated with 10 plaque-forming units/cell reovirus for 72 hours and cell death was evaluated using LIVE/DEAD flow cytometry, after gating on CTG^+^ MM cells. The percentage of viable cells is shown and after normalization to the untreated control for each co-culture condition. (B) Junctional adhesion molecule A (JAM-A) expression after co-culture on HS-5 or HS-27 BM stromal cells for 24 hours. Asterisk (*) denotes statistical significance and error bars indicate SEM for n=3 experiments. *P<0.05, **p<0.01.

In accordance with previous literature,^11^ we confirmed that human MM cell lines (H929, U266B and JIM3) expressed the reovirus entry receptor JAM-A; however, OPM2 cells were JAM-A negative (online supplemental figure S4A). Consistent with this, H929, U266B and JIM3 cell lines were susceptible to the direct lytic effects of reovirus, and OPM2 cells were resistant (online supplemental figure S4B). Nonetheless, in vivo, neoplastic MM cells do not exist in isolation and it is well recognized that the addition of BMSCs to hematopoietic neoplastic cells can provide a protective niche and induce a drug-resistant phenotype.^24^ Therefore, we co-cultured MM cells with BMSCs and examined the expression of the anti-apoptotic protein, Mcl-1, which is essential for the survival of malignant plasma cells in the bone marrow^25^; as expected, Mcl-1 was upregulated on co-culture with both HS-5 and HS-27 BMSCs.^26^ Next, we evaluated whether addition of BMSCs altered the susceptibility of MM cells to reovirus-direct oncolysis. Significantly, HS-27 BMSCs provided protection against reovirus oncolysis in H929, U266B and JIM3 MM cell lines, while HS-5 BMSCs protected H929 and JIM3 MM cells (figure 3A). Importantly, changes in reovirus susceptibility were not due to altered JAM-A expression as no significant changes in JAM-A expression were observed (figure 3B).

10.1136/jitc-2020-001803.supp5 Supplementary data

### Effect of BMSCs on reovirus-induced bystander cytokine killing

**Figure 4:**
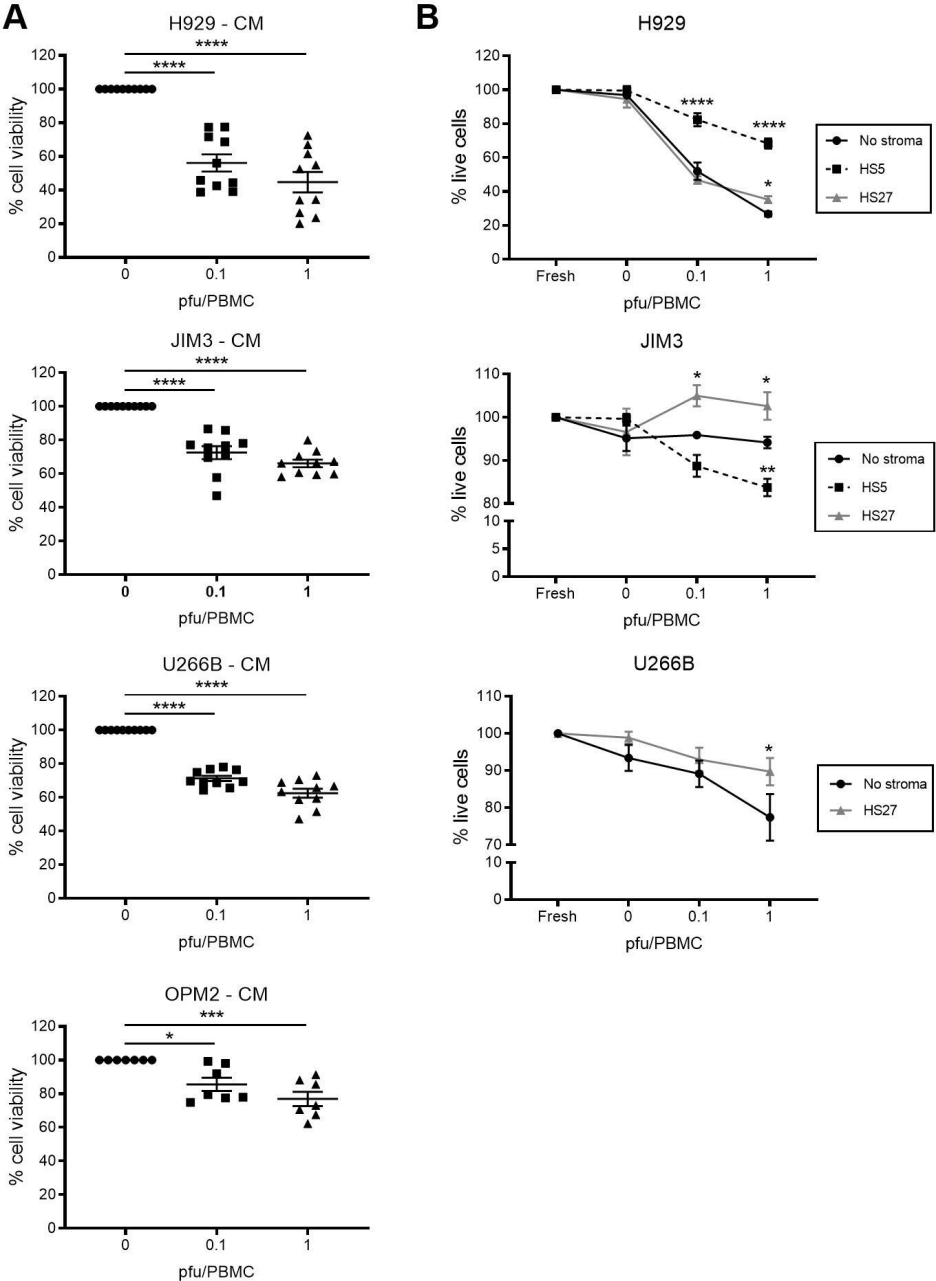
Bystander cytokine killing of human multiple myeloma (MM) cells and the effect of bone marrow stromal cells (BMSCs). Peripheral blood mononuclear cell-conditioned medium (PBMC-CM) was collected from untreated PBMC (0 plaque-forming units (PFU)/PBMC) or after treatment with reovirus (0.1 or 1 PFU/PBMC) for 48 hours and ultraviolet (UV)-irradiated to inactivate replication-competent reovirus. (A) H929, JIM3, U266B and OPM2 cells were cultured alone and treated with PBMC-CM for 96 hours and cell viability was determined by MTS. (B) H929, JIM3 and U266B (labeled with Cell Tracker Green (CTG)) were co-cultured with HS-5 or HS-27 BM stromal cells at a 1:1 ratio for 24 hours and treated with PBMC-CM for a further 72 hours. Cell death was evaluated using LIVE/DEAD flow cytometry, after gating on CTG^+^ MM cells; percentages were normalized to the untreated control for each co-culture condition. *P<0.05, **p<0.01, ***p<0.001, ****p<0.0001.

To date, reovirus-induced bystander cytokine killing has not been reported for MM; however, several cytokines have been implicated as a potential treatment, including type I and type II IFNs.^27 28^ Previous literature has reported the induction of type I IFN-α following reovirus treatment^16^ and here we have confirmed its secretion from healthy donor PBMC in response to reovirus treatment (0.1 or 1 PFU/PBMC) (online supplemental figure S5A). Moreover, on more detailed assessment of reovirus-induced cytokines using multiplex bead arrays we observed the induction of a diverse range of cytotoxic cytokines, including type II IFN-γ, TNF-α and TRAIL (online supplemental figure S5B). To examine reovirus-induced bystander cytokine killing, PBMC-CM was harvested (±reovirus treatment) and MM cells were cultured in PBMC-CM for 96 hours. PBMC-CM collected after reovirus treatment displayed cytotoxicity against both reovirus-resistant OPM2 cells and reovirus-susceptible H929, U266B and JIM3 cells (figure 4A). To confirm that the cytotoxicity was mediated by the presence of cytokines, the PBMC-CM was filtered to remove extracellular vesicles, which did not abrogate its cytotoxic effect (online supplemental figure S5C). Moreover, following treatment with recombinant cytokines, IFN-α (not TNF-α or IFN-γ) was implicated in the killing of H929 cells but not U266B cells, suggesting that additional, as yet undefined, cytokines are involved in mediating the overall cytotoxic effects of PBMC-CM (online supplemental figure S5D). Although we have not defined the role for individual cytokines in all cell lines, given our previous studies in different HM disease models,^6^ it is expected that multiple cytokines will contribute to this effect across different MM cell lines.

10.1136/jitc-2020-001803.supp6 Supplementary data

Interestingly, on examination of bystander cytokine killing in the context of BMSCs, some, although incomplete, reversal of killing was observed; co-culture with HS-5 BMSCs protected H929 cells from reovirus-conditioned PBMC-CM cytotoxicity and HS-27 BMSCs protected both U266B and JIM3 cells (figure 4B). Of note, it is postulated that the differential responses observed with JIM3 cells shown in figure 4A, B were due to the reduced exposure time used in figure 4B (72 hours instead of 96 hours), because of the 24 hours preconditioning period on BMSCs required prior to treatment with PBMC-CM. Nevertheless, it is also possible that PBMC-CM induces a cytostatic effect in JIM3 cells, which is detected by MTS assays (figure 4A) but not seen using the LIVE/DEAD assay (figure 4B). Taken together, these results demonstrate that the cytokine milieu induced by reovirus can have a toxic effect on MM cells, even when cells are resistant to reovirus oncolysis (ie, OPM2 cells). However, within the BM niche, MM cells can be protected from both direct reovirus oncolysis and bystander cytokine killing.

### Effect of BMSCs on reovirus-mediated NK cell killing

**Figure 5:**
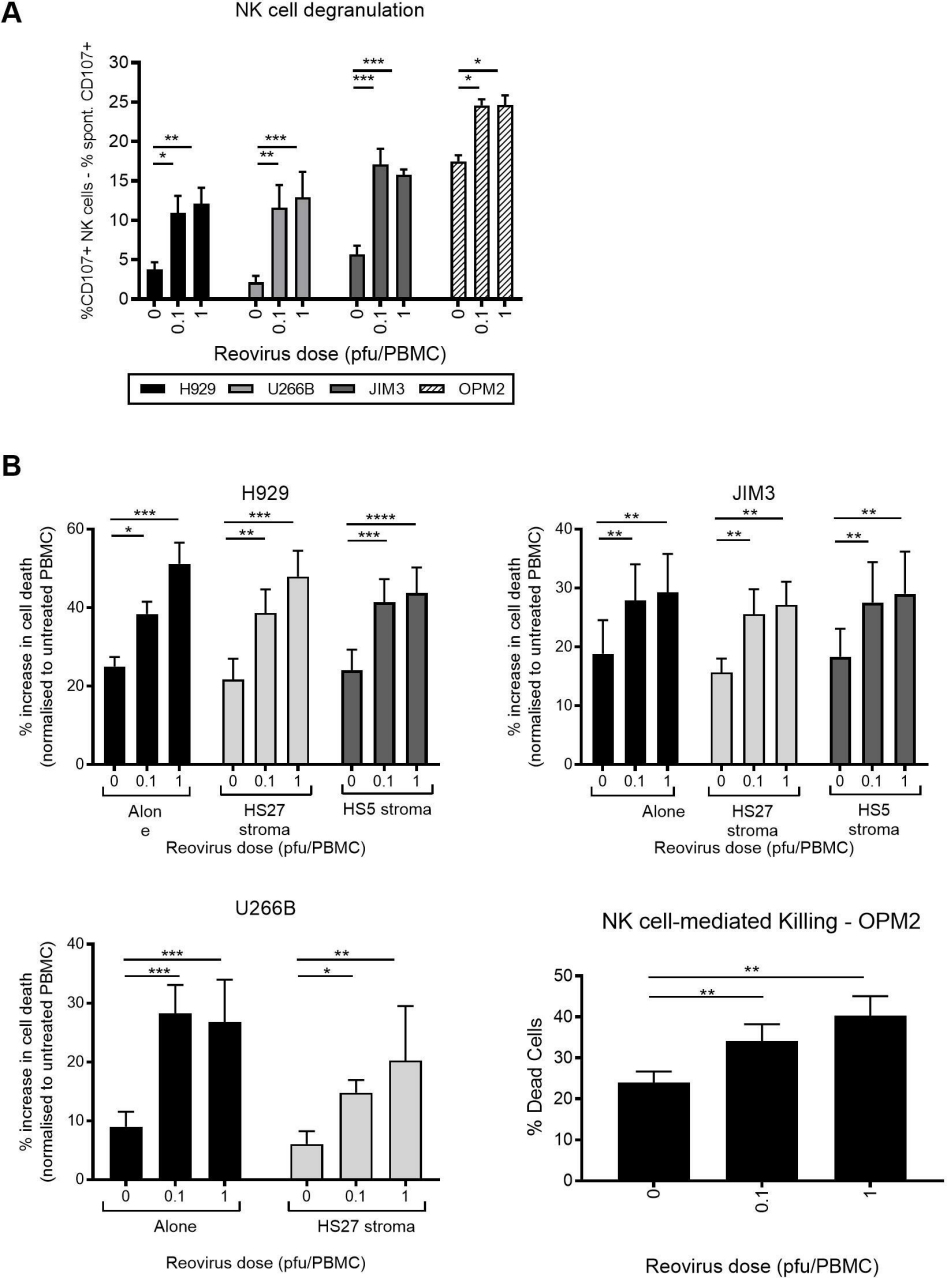
Reovirus-activated natural killer (NK) cells kill multiple myeloma (MM) cells which are resistant to reovirus-direct oncolysis. (A) Peripheral blood mononuclear cell (PBMCs) were treated with 0.1 or 1 plaque-forming units/cell reovirus for 48 hours and co-cultured with MM target cells at a 2:1 effector:target ratio for 1 hour, followed by a further 4 hours with brefeldin A. CD107a/b expression on NK cells within the PBMC population (±reovirus treatment) following co-culture with H929, U266B, JIM3 or OPM2 cells is shown. (B) Cell Tracker Green (CTG)^+^ MM target cells (±co-cultured with HS-5 or HS-27 BMSCs for 48 hours) were co-cultured with PBMC (±reovirus treatment) at a 25:1 effector:target ratio for 5 hours. The percentage cell death of CTG^+^ MM cells was determined using LIVE/DEAD. Error bars indicate SEM for n=4 independent experiments and asterisk (*) denote statistical significance. *P<0.05, **p<0.01, ***p<0.001, ****p<0.0001.

As demonstrated by the data presented so far, the BM niche can protect MM cells from direct reovirus oncolysis and cytokine-mediated killing. However, activation of immune effector cells (specifically, NK cells and CD8^+^ T cells) within the BM of reovirus-treated animals (figure 2D) suggests additional effector mechanisms that may be capable of overcoming this resistance phenotype. In accordance with previous literature,^16^ NK cells significantly upregulated CD69 following reovirus treatment of PBMC (data not shown). Moreover, enhanced NK cell degranulation against MM cell targets (figure 5A) and NK cell-mediated death was observed after reovirus treatment (figure 5B). Overall, the ability of reovirus-activated NK cells to kill MM cells was not significantly abrogated on co-culture with BMSCs (except for U266B cells at lower reovirus doses), and importantly, NK cell-mediated killing of reovirus-resistant OPM2 cells was also observed (figure 5B). Consistent with these findings, the expression of NK cell activatory ligands on MM cells did not change following BMSC co-culture (online supplemental figure S6). These results demonstrate that reovirus-activated NK cells can effectively kill MM cells that may be protected from reovirus-direct oncolysis within the BM niche.

10.1136/jitc-2020-001803.supp7 Supplementary data

### Reovirus-induced MM-specific CTLs

**Figure 6:**
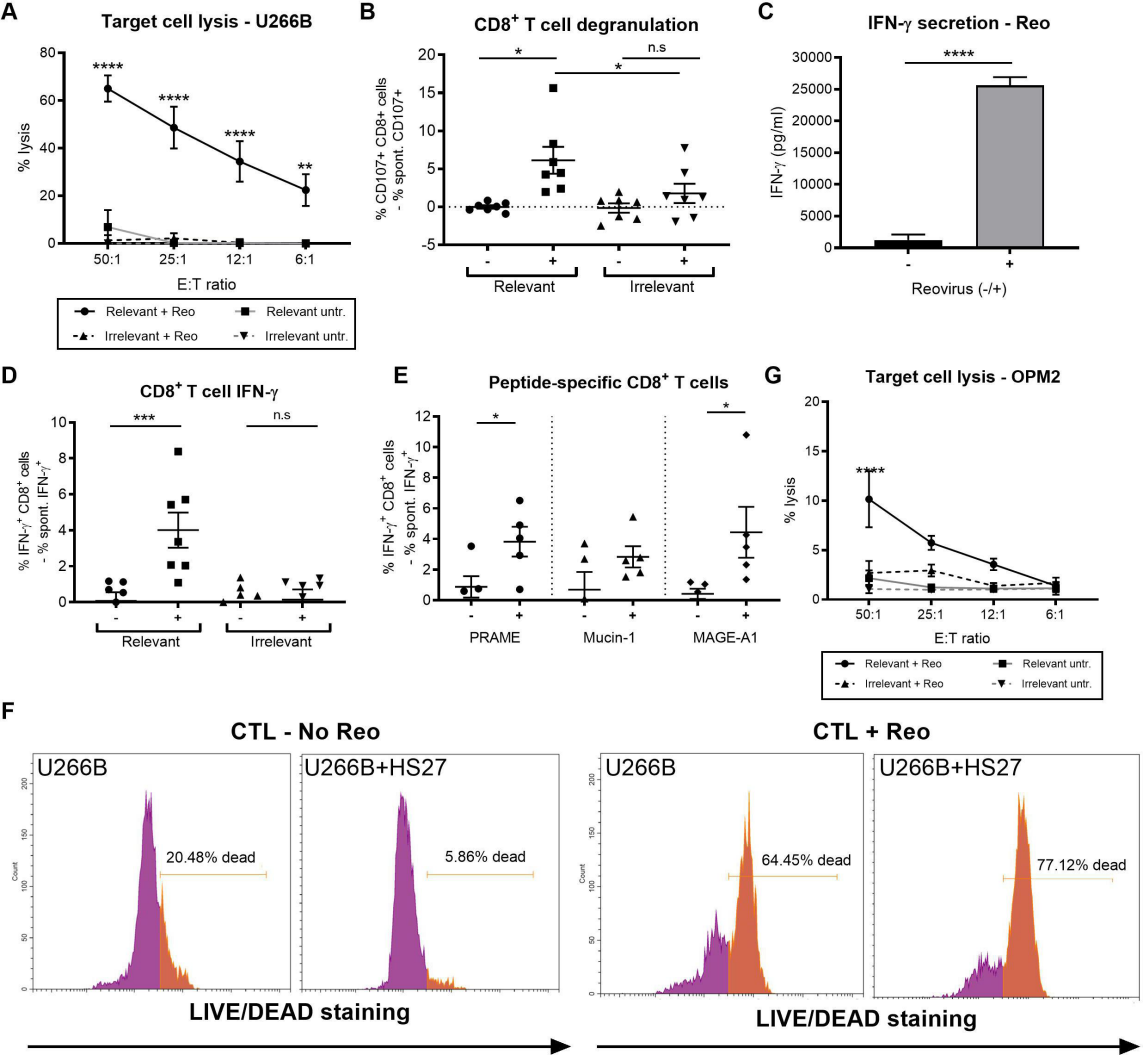
Reovirus treatment of multiple myeloma (MM) cells stimulates the production of MM-specific cytotoxic T lymphocytes (CTLs). (A) CTLs were generated by co-culturing peripheral blood mononuclear cells (PBMC) with autologous dendritic cell (DC), preloaded with reovirus-sensitive U266B (±1 plaque-forming units (PFU)/cell reovirus). CTLs generated were co-cultured with ^51^Cr-labeled relevant (U266B) and irrelevant (KG-1) target cells and the percentage cell lysis was determined after 4 hours using ^51^Cr assays (n=6). (B) Reovirus-primed CTLs were co-cultured with relevant (U266B) or irrelevant (KG-1) target cells at a 2:1 effector:target ratio and CD107a/b expression on CD3^+^CD8^+^ CTL was determined (n=7). (C) Interferon (IFN)-γ levels in CTL priming cultures was determined by ELISA (n=6). (D) CTLs generated ±reovirus were co-cultured with relevant (U266B) or irrelevant (KG-1) target cells at a 2:1 effector:target ratio for 1 hour, followed by a further 4 hours with brefeldin A. CTLs were fixed and permeabilized prior to intracellular IFN-γ quantification (n=7). (E) CTLs generated ±reovirus were co-cultured with autologous CD14^+^ cells loaded with PRAME, mucin-1 and MAGE-A1 peptide pools (1 hour at 37°C). Cells were co-cultured at a 2:1 effector:target ratio and intracellular IFN-γ was quantified (n=5). (F) CTLs were co-cultured with CTG-labeled U266B target cells alone, or CTG-labeled U266B cells precultured on HS-27 stromal cells for 48 hours, at a 25:1 effector:target ratio. Cell death of CTG^+^ U266B cells was determined using LIVE/DEAD. Representative flow cytometry plots are shown and the percentage of dead cells (orange gate) is indicated in each plot. Left plots: U266B targets alone. Right plots: U266B cells precultured with HS-27 stromal cells. (G) CTLs were generated by co-culturing PBMC with autologous DC, preloaded with reovirus-resistant OPM2 cells (±1 PFU/cell reovirus). CTLs generated were co-cultured with ^51^Cr-labeled relevant (OPM2) and irrelevant (KG-1) target cells and the percentage cell lysis was determined after 4 hours using ^51^Cr assays (n=4). Asterisk (*) denotes statistical significance and error bars indicate SEM. *P<0.05, **p<0.01, ***p<0.001, ****p<0.0001.

Activation of CD8^+^ T cells in vivo following reovirus delivery, and the negative correlation between CD8^+^ T_EM_ cells and tumor burden (figure 2), suggested a potential role for T cells in reovirus efficacy; therefore, we sought to examine this using human CTL priming assays.^6^ As expected, reovirus treatment of human iDC increased expression of CD80, CD86 and HLA-DR on DC (online supplemental figure S7A),^18^ demonstrating its potential to support priming of adaptive MM-specific CTLs. However, to test this, iDC were loaded with reovirus-treated U266B cells and cultured with autologous PBMC over the course of 3 weeks. Primed CTLs cells were subsequently evaluated for their tumor specificity using relevant U266B MM targets or irrelevant KG-1 controls. Figure 6A demonstrates that CTLs generated in the presence of reovirus specifically kill U266B targets, with no enhanced killing of irrelevant KG-1 cells; moreover, CTL degranulation was only observed against U266B targets, not irrelevant KG-1 controls (figure 6B), and was completely abrogated when major histocompatibility complex (MHC) class I was blocked (online supplemental figure S7B), confirming the importance of MHC class I–CD8 interactions.

10.1136/jitc-2020-001803.supp8 Supplementary data

To further characterize the CTL response, the production of IFN-γ—secreted in large amounts on antigen recognition by CD8^+^ CTLs and Th1 CD4^+^ T cells^29^—was examined. ELISA assays confirmed abundant secretion of IFN-γ in reovirus-primed CTL cultures, suggestive of a Th1-skewed immune response (figure 6C).^29^ Furthermore, assessment of IFN-γ using intracellular flow cytometry demonstrated that CD8^+^ CTLs (primed using reovirus-loaded U266B cells) secreted IFN-γ on recognition of relevant (U266B), but not irrelevant (KG-1) cells (figure 6D). Subsequently, CTLs were challenged with peptide pools of TAA (PRAME, mucin-1 and MAGE-A1), which are commonly expressed in MM,^30^ and increased IFN-γ production was observed in response to PRAME and MAGE-A1 peptides (figure 6E). As expected, CTL responses were variable between donors, and although not significant, IFN-γ production was also induced following stimulation with mucin-1 peptides in some donors (figure 6E). Having confirmed that reovirus can prime CTL responses against well-recognized and reported TAA, we next explored the ability of CTLs to kill MM in the presence of BMSC support. CTL priming assays were performed, as above, and the ability of reovirus-primed CTLs to kill U266B cells alone, or U266B co-cultured with HS-27 BMSCs (which inhibited reovirus oncolysis), was evaluated. Encouragingly, there was no significant reduction in the killing of U266B target cells with prior co-culture on BMSCs (Figure 6F and online supplemental figure S7C).

Importantly, MM-specific CTLs could also be generated using an alternative reovirus-sensitive MM cell line, H929 (online supplemental figure S7Di) and, as observed for U266B-specific CTLs, H929-generated CTLs had the capacity to kill H929 cells cultured with or without BMSCs (online supplemental figure S7Dii). Moreover, although CTL priming was less efficient when using reovirus-resistant OPM2 MM cells (figure 6G), reovirus was also capable of generating OPM2-specific CTLs. Taken together, these findings demonstrate that reovirus treatment of MM cells can generate MM-specific CTLs capable of killing MM cells, even when they reside in a protective BM niche.

## Discussion

MM remains an incurable malignancy despite a raft of new therapeutic agents, thus, novel therapeutic strategies are being actively sought. Previous work demonstrated that MM cells are susceptible to the direct lytic effects of reovirus^13^ and that reovirus can reduce MM tumor burden, in vivo.^31^ However, building on these observations, we have shown that reovirus treatment decreases both tumor burden and MBD, and that while human MM cell lines (except OPM2) are susceptible to reovirus direct oncolysis, permissiveness to infection is reduced on co-culture with BMSCs. To our knowledge, this is the first time that BMSCs have been reported to protect cells from direct OV oncolysis. Indeed, co-culture of pancreatic cancer cells with cancer-associated fibroblasts (CAF) can potentiate viral propagation in both cancer cells and CAF.^32^ The mechanism underpinning reovirus-resistance remains unknown, although one possible explanation is upregulation of Mcl-1^26^ and subsequent resistance to reovirus-induced apoptosis.^9 33^

A range of immune effects are induced by reovirus,^16 18^ therefore, it was possible that immune-mediated mechanisms could circumvent stromal-mediated resistance. When immune subsets were examined in our murine model, reovirus treatment altered the immune profile and induced lymphocyte activation. A significant increase in T_EM_ following reovirus treatment, and concordant decrease in naïve T cells, is characteristic of long-term immunological memory following antigen recognition^34 35^; moreover, the induction of antitumor immunity was further supported by the increase in PD-1 on CD4 and CD8 T cells, the presence of 5TGM1-specific CD8^+^ cells and the negative correlation of CD8^+^ T_EM_ cells with tumor burden. Interestingly, we were unable to detect reovirus in the BM of reovirus-treated animals on sacrifice, suggesting that reovirus does not persist in the BM after repeated administrations; early reovirus replication/oncolysis could play a role in the induction of antitumor immunity or reovirus may promote antitumor immune responses in the absence of oncolysis, a phenomena that has been previously reported.^36^

Here, we demonstrate that reovirus-induced antimyeloma activity engages both innate and adaptive arms of the immune system encompassing: (1) cytokine-mediated bystander killing; (2) NK cell-mediated cellular cytotoxicity and (3) priming of MM-specific CTLs. Differential effects on direct oncolysis and bystander cytokine killing were observed on co-culture with HS-5 or HS-27 BMSCs. While the reason for this warrant’s further investigation, the most likely explanation is the intrinsic properties of the BMSCs used; HS-5 cells produce a range of growth factors/cytokines to support MM cell survival, while HS-27 cells rely on cell-to-cell interactions.^37^ It is therefore possible that the cytokines secreted from HS-5 cells could potentiate OV-induced cytokine killing (as seen for JIM-3), although this would be dependent on the repertoire of growth factor/cytokine receptors available. Indeed, differential sensitivity of JIM-3 and H929 MM cells to anti-IGF-1 receptor antibodies has been reported—with enhanced sensitivity in JIM-3—demonstrating the distinct surface phenotypes of different MM cell lines.^38^ Furthermore, data presented in online supplemental figure S4A, and in our previously published work,^6^ showed that JIM-3 and U266B cells express higher levels of JAM-A and intercellular adhesion molecule-1 (important for cell adhesion) than other MM cell lines, therefore, enhanced cell-to-cell interactions may make these cells more amenable to HS-27 BMSC support.

Previously, the ability of activated NK cells to kill MM target cells, including reovirus-resistant OPM2 cells, has been reported.^39^ Importantly, we have expanded these observations and demonstrated that reovirus-activated NK cells can kill MM cells, irrespective of BM stromal support. NK cells (and indeed CTLs) kill target cells via the release of cytotoxic granules that contain perforin and multiple granzymes. Granzyme B (GrB), commonly expressed by NK cells and CTLs, uses multiple pathways to induce apoptosis,^40^ therefore modulation of distinct pathways by BMSCs may be sufficient to inhibit reovirus oncolysis and OV-induced bystander cytokine killing, but insufficient to resist the multimodal action of GrB.

Importantly, reovirus-activated NK cells are specific for malignant cells and do not degranulate against non-malignant hepatocytes^41^ or other immune cell populations present in mixed PBMC cultures (data not shown), in keeping with the excellent safety profile of reovirus in clinical trials.^42^ Moreover, while the human in vitro system used in this study involved allogeneic MM cell lines, we have previously confirmed that NK cells can degranulate against autologous tumor cells,^16^ and here we show that murine NK cells degranulate against syngeneic 5TGM1 cell targets (online supplemental figure S3D). Furthermore, cell death within 4 hours is characteristic of NK cell-mediated killing on addition of PBMC and we have previously validated the role for NK cells in these well-defined in vitro assays.^41 43 44^ Collectively, our data are consistent with a therapeutic role for reovirus-activated NK cells and is in agreement with previous work using alternative cancer models.^45 46^

Adaptive T cell antitumor immunity is required for the generation of long-term immunological memory. Therefore, the observation that primed CTLs were effective at killing MM targets—irrespective of BM stromal support—is significant, as is the fact that the CTLs generated had specificity towards known TAAs. Interestingly, we were also able to prime CTLs against reovirus-resistant OPM2 cells, suggesting that direct oncolysis was not required, consistent with our previous work using UV-inactivated reovirus which primed melanoma-specific CTLs comparable to replication-competent reovirus.^36^ With regard to the priming of OPM2-specific CTLs, both U266B and OPM2 express PRAME and MAGE-A1,^47^ TAA that were recognized by U266B primed-CTLs, therefore, OPM2-primed CTLs could share specificity towards the same myeloma-associated TAA.

## Conclusions

Our data suggest that induction of antitumor immunity by an OV can overcome stromal-mediated tumor resistance—a key mechanism by which MM cells escape chemotherapeutic control and give rise to relapse. Here, we delineate a combination of innate and adaptive mechanisms for immune control of the disease; therefore, rationale combination approaches to optimize these approaches are required. Furthermore, therapeutic intervention should be considered when disease burden is low and immune function is relatively preserved (eg, in remission after autologous stem cell transplant or during maintenance therapy) to eliminate low-level residual disease protected within the BM niche.

10.1136/jitc-2020-001803.supp3 Supplementary data
