# Supplementary material for: Reovirus-induced cell-mediated immunity for the treatment of multiple myeloma within the resistant bone marrow niche
Source: J Immunother Cancer. 2021 Mar 19;9(3):e001803. doi: 10.1136/jitc-2020-001803 (PMC7986878; doi:10.1136/jitc-2020-001803)
Supplement: Supplementary data [file jitc-2020-001803supp001.pdf]

Supplementary Table S1: Murine flow cytometry antibodies.

Target molecule	Fluorochrome	Volume added	Species of origin	Clone	Supplier
**CD138**	PE	2 µL	REA	REA104	Miltenyi Biotec
**CD3**	PE-Vio770	2 µL	Hamster	145-2C11	Miltenyi Biotec
**DX5 (CD49b)**	PE	2 µL	Rat	R1-2	Miltenyi Biotec
**CD4**	VioBlue	2 µL	Rat	GK1.5	Miltenyi Biotec
**CD8**	PerCP	2 µL	Rat	53-6.7	Miltenyi Biotec
**CD44**	FITC	2 µL	Rat	IM7.8.8	Miltenyi Biotec
**CD62L**	APC	2 µL	Rat	MEL14-H2.100	Miltenyi Biotec
**CD69**	FITC	2 µL	Hamster	H1.2F3	Miltenyi Biotec

PE: phycoerythrin, PerCP: peridinin chlorophyll protein complex, FITC: fluorescein isothiocyanate: APC: Allophycocyanin REA: recombinant antibody

Supplementary Table S2: Human flow cytometry antibodies.

Target molecule	Fluorochrome	Volume added	Species of origin	Clone	Supplier
**NK cell activation**					
**CD3**	PerCP	5 µL	Mouse	SP34-2	BD Biosciences
**CD56**	PE	2 µL	Mouse	AF13-7H3	Miltenyi Biotec
**CD69**	FITC	5 µL	Mouse	L78	BD Biosciences
**IgG_1**	FITC	5 µL	Mouse	MOPC-21	BD Biosciences
**NK cell degranulation**					
**CD3**	PerCP	5 µL	Mouse	SP34-2	BD Biosciences
**CD56**	PE	2 µL	Mouse	AF13-7H3	Miltenyi Biotec
**CD107a**	FITC	5 µL	Mouse	H4A3	BD Biosciences
**CD107b**	FITC	5 µL	Mouse	H4B4	BD Biosciences
**CTL degranulation**					
**CD3**	VioBlue	2 µL	Mouse	BW264/56	Miltenyi Biotec
**CD56**	PE	2 µL	Mouse	AF13-7H3	Miltenyi Biotec
**CD8**	PerCP	2 µL	Mouse	BW135/80	Miltenyi Biotec
**CD107a**	FITC	5 µL	Mouse	H4A3	BD Biosciences
**CD107b**	FITC	5 µL	Mouse	H4B4	BD Biosciences

Target molecule	Fluorochrome	Volume added	Species of origin	Clone	Supplier
**NK ligand phenotyping**					
**MIC A/B**	PE	5 µL	Mouse	6D4	BD Biosciences
**ULBP-1**	PE	5 µL	Mouse	#170818	R&D Systems
**ULBP-2/5/6**	PE	5 µL	Mouse	#165903	R&D Systems
**PVR**	PE	5 µL	Mouse	PV404.19	Miltenyi Biotec
**Nectin-2**	PE	5 µL	Mouse	R2.525	BD Biosciences
**HLA A/B/C**	PE	5 µL	Mouse	EMR8-5	BD Biosciences
**IgG_{2a}**	PE	5 µL	Mouse	G155-178	BD Biosciences
**IgG_{2b}**	PE	5 µL	Mouse	27-35	BD Biosciences
**Virus entry receptor phenotyping**					
**JAM-A**	PE	5 µL	Mouse	M.Ab.F11	BD Biosciences
**IgG_1**	PE	5 µL	Mouse	H4B4	BD Biosciences
**Intracellular IFN-γ staining (CTL)**					
**CD3**	VioBlue	2 µL	Mouse	BW264/56	Miltenyi Biotec
**CD56**	PE	2 µL	Mouse	AF13-7H3	Miltenyi Biotec
**CD8**	PerCP	2 µL	Mouse	BW135/80	Miltenyi Biotec
**IFN-γ**	FITC	7 µL	Mouse	45-15	Miltenyi Biotec

PE: phycoerythrin, PerCP: peridinin chlorophyll protein complex, FITC: fluorescein isothiocyanate.
