# Supplementary material for: Reovirus-induced cell-mediated immunity for the treatment of multiple myeloma within the resistant bone marrow niche
Source: J Immunother Cancer. 2021 Mar 19;9(3):e001803. doi: 10.1136/jitc-2020-001803 (PMC7986878; doi:10.1136/jitc-2020-001803)
Supplement: Supplementary data [file jitc-2020-001803supp002.pdf]

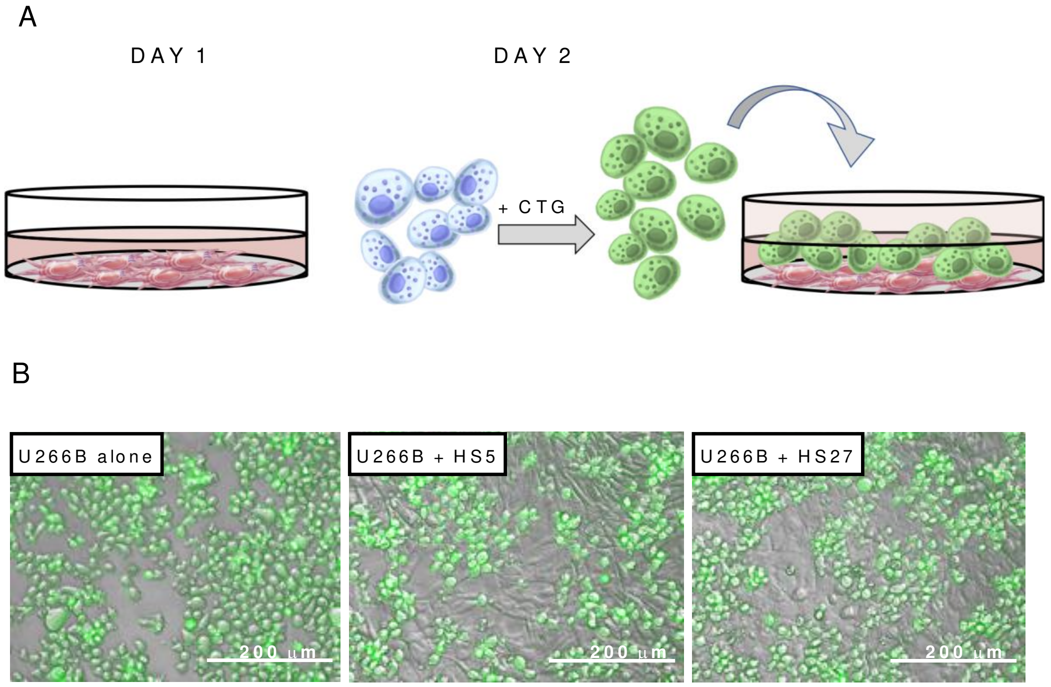

Supplementary Figure 1: MM:BMSC co-cultures. A) On Day 1, stromal cells (HS-5 or HS-27) were seeded and allowed to adhere. On Day 2, MM cells were stained with Cell Tracker Green (CTG) and added to stromal cells at a 1:1 ratio. Any medium already in the culture wells (conditioned by the stromal cells) was diluted 1:1 with fresh medium upon the addition of MM cells. B) Representative images of U266B cells cultured alone, or together with HS-5, or HS-27 stromal cells, respectively. Images taken using an EVOS® FL Cell Imaging System after a 24 hrs co-culture (ThermoFisher Scientific).
