# Supplementary material for: Reovirus-induced cell-mediated immunity for the treatment of multiple myeloma within the resistant bone marrow niche
Source: J Immunother Cancer. 2021 Mar 19;9(3):e001803. doi: 10.1136/jitc-2020-001803 (PMC7986878; doi:10.1136/jitc-2020-001803)
Supplement: Supplementary data [file jitc-2020-001803supp004.pdf]

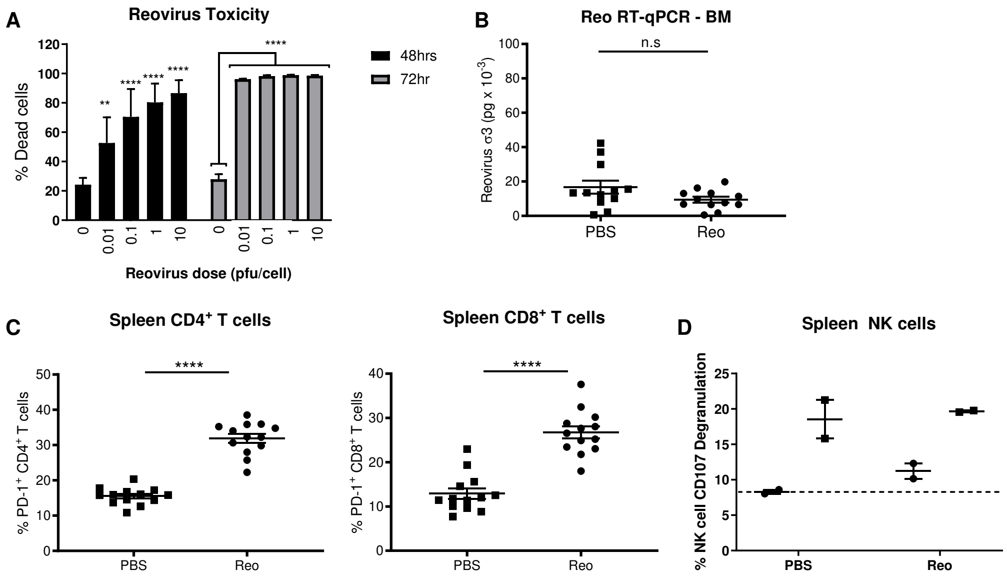

Supplementary Figure 3: Susceptibility of 5TGM1 cells to reovirus e*x vivo* and characterisation of reovirus responses *in vivo*. A. 5TGM1 cells were either left untreated (0pfu/cell) or treated with reovirus (0.01, 0.1, 1, or 10 pfu/cell) for 48 or 72 hrs (n=3). Cell viability was measured at each time point using Live/Dead®. *denotes statistical significance compared to untreated cells. Error bars indicate SEM. **B.** The presence of reovirus in the BM of control (PBS treated) or reovirus-treated animals (upon termination) was examined by RT-qPCR using reovirus σ3 primers (n=12 per group). RNA was isolated using the RNEasy Mini Kit and results were quantified using a standard curve method. Error bars indicate SEM and statistical significance was calculated using two-tailed, unpaired *t*-tests; n.s = not significant. **C.** At sacrifice, the percentage of $PD\text{-}1^+CD4^+$ and $PD\text{-}1^+CD8^+$ T cells was quantified in the spleen (n=13). Error bars indicate SEM and *denotes statistical significance **D**. 96hrs post treatment, splenocytes were isolated and co-cultured with 5TGM1 cells *ex vivo* for 4 hours in the presence of brefeldin A; the percentage of DX5+NK cells expressing CD107 was determined by flow cytometry.
