# Supplementary material for: Reovirus-induced cell-mediated immunity for the treatment of multiple myeloma within the resistant bone marrow niche
Source: J Immunother Cancer. 2021 Mar 19;9(3):e001803. doi: 10.1136/jitc-2020-001803 (PMC7986878; doi:10.1136/jitc-2020-001803)
Supplement: Supplementary data [file jitc-2020-001803supp005.pdf]

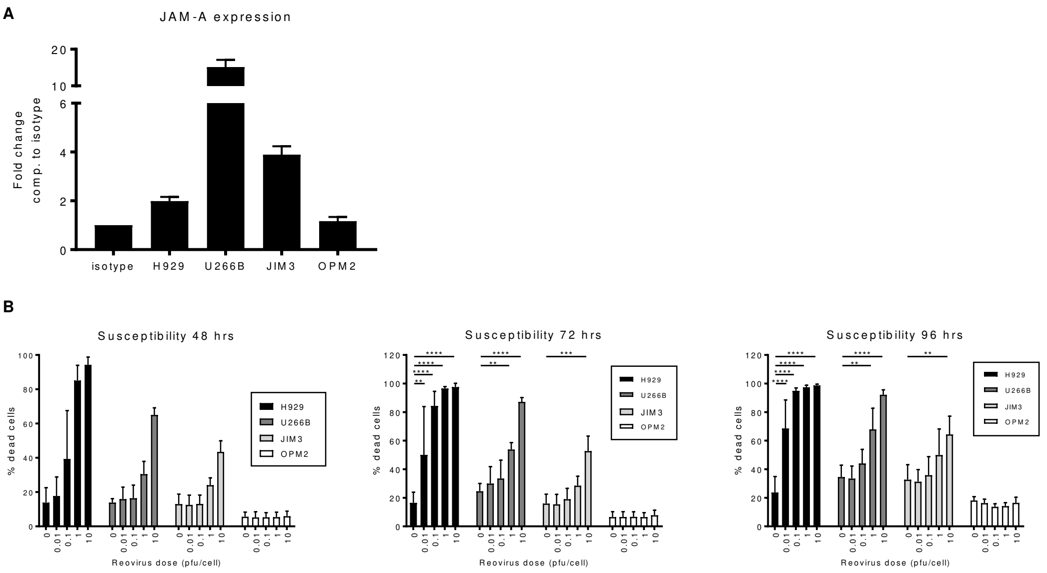

Supplementary Figure 4: Expression of JAM-A on human MM cell lines and susceptibility to reovirus oncolysis. A. MM cell lines H929, U266B, JIM3, and OPM2 were phenotyped for baseline expression of JAM-A. Fold change in mean fluorescence intensity (MFI) compared to an isotype control is shown (n=3). **B.** All cell lines were left untreated (0 pfu/cell) or treated with either 0.01, 0.1, 1, or 10 pfu/cell reovirus for 48, 72 or 96 hrs. Cell death was determined using a Live/Dead® discrimination stain (n=3). *denotes statistical significance and error bars indicate SEM.
