# Supplementary material for: Reovirus-induced cell-mediated immunity for the treatment of multiple myeloma within the resistant bone marrow niche
Source: J Immunother Cancer. 2021 Mar 19;9(3):e001803. doi: 10.1136/jitc-2020-001803 (PMC7986878; doi:10.1136/jitc-2020-001803)
Supplement: Supplementary data [file jitc-2020-001803supp006.pdf]

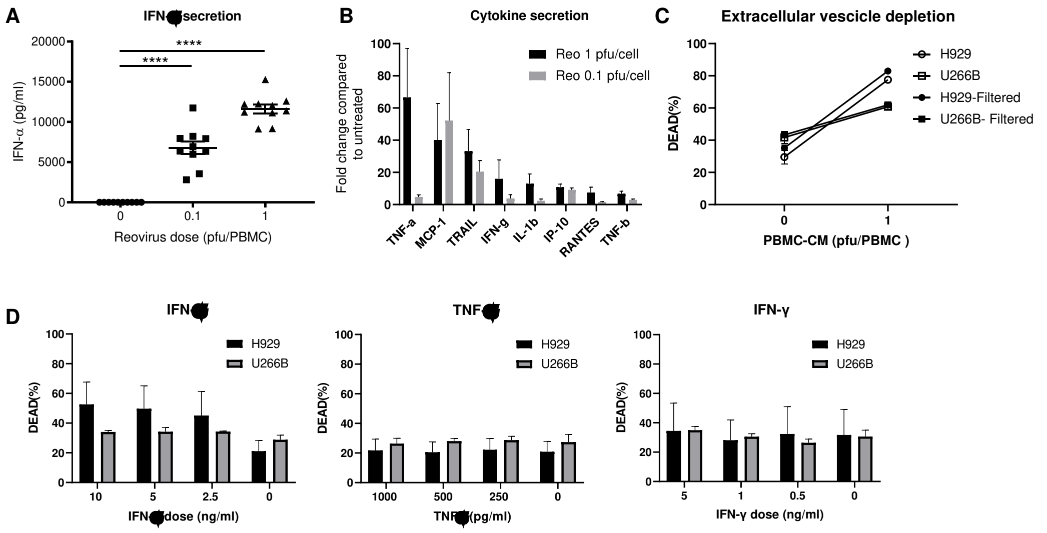

Supplementary Figure 5: Cytokine secretion from healthy donor PBMC following reovirus treatment and characterisation of PBMC-CM mediated killing. Healthy donor PBMC were treated for 48 hrs with 0.1 or 1 pfu/cell of reovirus (n=10) and cell-free supernatants were harvested. **A.** Secretion of IFN-α was measured by ELISA. **B.** Pro-inflammatory cytokine secretion was determined using multiplex bead arrays; data shows the fold increase in cytokines compared to untreated PBMC +SEM for n=3 donors. *denotes statistical significance and error bars indicate SEM. **C.** PBMC-CM was filtered to remove extracellular vesicles and cytotoxicity against H929 and U266B cells was determined using LIVE/DEAD. **D.** H929 and U266B cells were treated with increasing doses of recombinant cytokines and cell death was determined using LIVE/DEAD.
