# Supplementary material for: Reovirus-induced cell-mediated immunity for the treatment of multiple myeloma within the resistant bone marrow niche
Source: J Immunother Cancer. 2021 Mar 19;9(3):e001803. doi: 10.1136/jitc-2020-001803 (PMC7986878; doi:10.1136/jitc-2020-001803)
Supplement: Supplementary data [file jitc-2020-001803supp007.pdf]

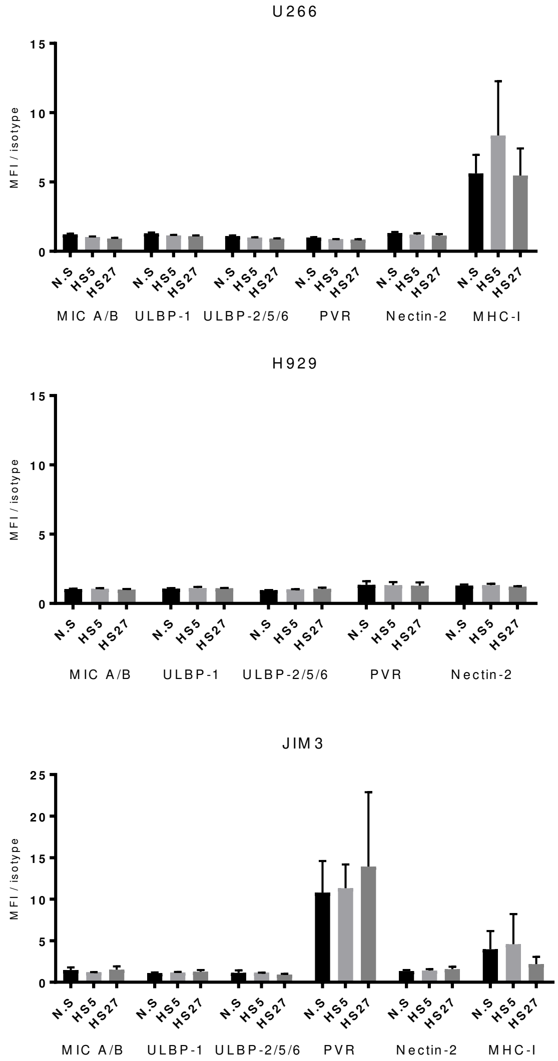

Supplementary Figure 6: **NK cell activatory ligands do not change following co-culture with BMSCs.** U266B, H929 and JIM3 were either cultured alone or co-cultured with HS5 or HS27 BMSCs for 48hrs and the expression of NK cell activatory ligands was determined by flow cytometry. Error bars show the mean of at least n=2 independent experiments.
