# Supplementary material for: Reovirus-induced cell-mediated immunity for the treatment of multiple myeloma within the resistant bone marrow niche
Source: J Immunother Cancer. 2021 Mar 19;9(3):e001803. doi: 10.1136/jitc-2020-001803 (PMC7986878; doi:10.1136/jitc-2020-001803)
Supplement: Supplementary data [file jitc-2020-001803supp008.pdf]

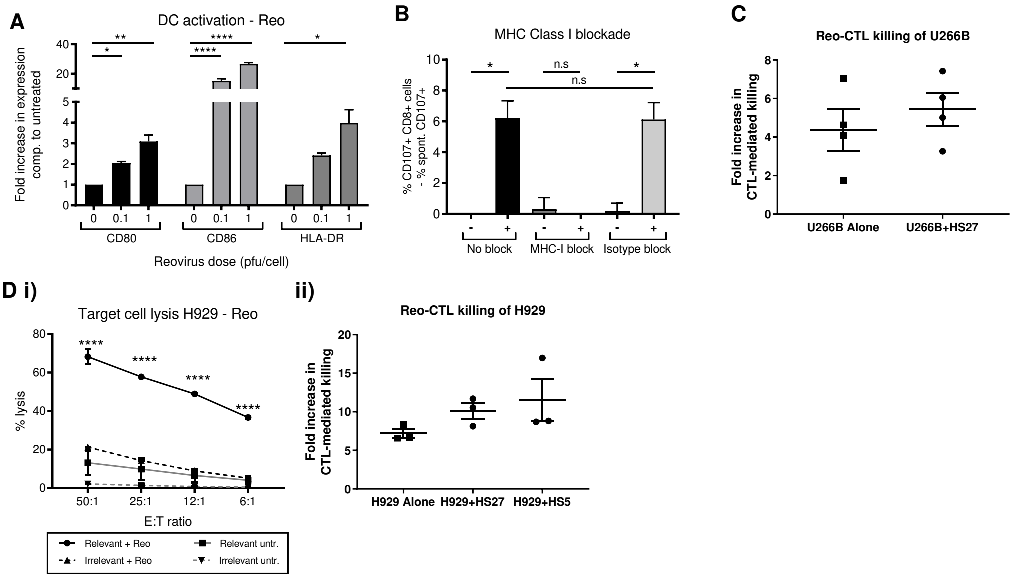

Supplementary Figure 7: Generation of MM-specific CTLs using reovirus. **A.** Immature DCs were differentiated from CD14$^+$ cells obtained from healthy donor PBMC. Following differentiation, DC were treated with 0.1 or 1 pfu/cell reovirus for 24 hrs and expression of CD80, CD86, and MHC Class II (HLA-DR) was evaluated by flow cytometry. Fold increase in MFI compared to untreated DCs is shown (n=4). **B.** U266B targets were pre-labelled with an MHC Class I-blocking antibody or isotype control for 30min at 37°C and CTL degranulation was examined (n=3). **C.** CTLs were primed using sheared reovirus-treated U266B cells and co-cultured at a 25:1 effector:target ratio with CTG-labelled U266B cells alone, or CTG-labelled U266B cells pre-cultured on HS-27 stromal cells for 48 hrs. Cell death of CTG$^+$ U266B cells was determined using Live/Dead®; fold increase in cell death with reovirus-primed CTLs over no CTLs is shown. **D. i)** CTL priming was performed using reovirus-treated H929 cells (1pfu/cell) and the ability of CTLs to kill relevant (H929) and irrelevant (KG-1) target cells was evaluated by ^{51}Cr release (n=3). **ii)**. CTLs primed using sheared reovirus-treated H929 cells were co-cultured at a 25:1 effector:target ratio with CTG-labelled H929 target cells alone, or CTG-labelled H929 cells pre-cultured on HS-27 or HS-5 stromal cells for 48 hrs. Cell death of CTG$^+$ H929 cells was determined using Live/Dead®; fold increase in cell death with reovirus-primed CTLs over no CTLs is shown. Error bars indicate SEM and *denotes statistical significance.
