# Supplementary material for: Reovirus-induced cell-mediated immunity for the treatment of multiple myeloma within the resistant bone marrow niche
Source: J Immunother Cancer. 2021 Mar 19;9(3):e001803. doi: 10.1136/jitc-2020-001803 (PMC7986878; doi:10.1136/jitc-2020-001803)
Supplement: Supplementary data [file jitc-2020-001803supp003.pdf]

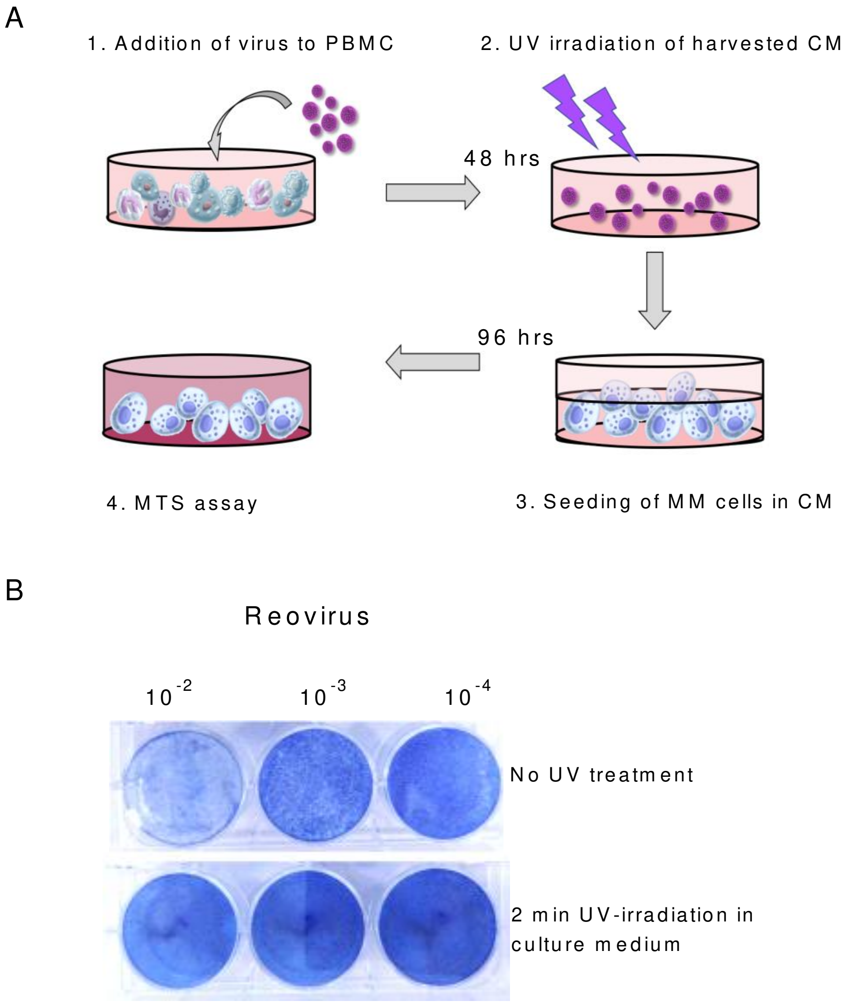

Supplementary Figure 2: UV inactivation of reovirus. A) CM was harvested from PBMC treated with either 0, 0.1, or 1 pfu/PBMC of reovirus for 48 hrs (1) and UV-irradiated for 2 min (2). MM Cells were then resuspended in CM diluted 1:1 in fresh culture medium (3) and after 96 hrs incubation cell viability was measured by MTS assays (4). B) Representative images showing reovirus plaques (monolayer destruction) when L929 cell were treated with different dilutions of stock reovirus, ±UV-irradiation for 2 minutes.
